# Visualization of the 3D structures of small organisms via LED-SIM

**DOI:** 10.1186/s12983-016-0158-9

**Published:** 2016-06-14

**Authors:** Yongying Ruan, Dan Dan, Mengna Zhang, Ming Bai, Ming Lei, Baoli Yao, Xingke Yang

**Affiliations:** Key Laboratory of Zoological Systematics and Evolution, Institute of Zoology, Chinese Academy of Sciences, Beijing, 100101 China; State Key Laboratory of Transient Optics and Photonics, Xi’an Institute of Optics and Precision Mechanics, Chinese Academy of Sciences, Xi’an, 710119 China; University of Chinese Academy of Sciences, Beijing, 100039 China

**Keywords:** 3D imaging, Micro-structures, Structured illumination microscopy, LED illumination, Microscopy, Morphology

## Abstract

**Background:**

Innovative new techniques that aid in the visualization of microscopic anatomical structures have improved our understanding of organismal biology significantly. It is often challenging to observe internal 3D structures, despite the use of techniques such as confocal laser scanning microscopy (CLSM), micro-computed tomography (Micro-CT), magnetic resonance imaging (MRI), focused ion beam scanning electron microscopy (FIB-SEM) and others. In the current paper, we assess LED-SIM (DMD-based LED-illumination structured illumination microscopy), which facilitates the acquisition of nano- and micro-3D structures of small organisms in a high-resolution format (500 nm in the XY-plane and 930 nm along the Z-axis).

**Results:**

We compare other microstructural imaging techniques (involving conventional optical microscopy, CLSM and Micro-CT) with LED-SIM to assess the quality (e.g. resolution, penetration depth, etc.) of LED-SIM images, as well as to document the potential short-comings of LED-SIM. Based on these results we present an optimized set of protocols to ensure that LED-SIM arthropod and nematode samples with different cuticles or textures are prepared for analysis in an optimal manner. Six arthropod and nematode specimens were tested and shown to be suitable for LED-SIM imaging, which was found to yield high resolution 3D images.

**Conclusions:**

Although LED-SIM still must be thoroughly tested before it is widely accepted and the Z-axis resolution still requires improvement, this technique offers distinct high quality 3D images. LED-SIM can be highly effective and may provide high quality 3D images for zoological studies following the guidelines of sample preparation presented in the current paper.

**Electronic supplementary material:**

The online version of this article (doi:10.1186/s12983-016-0158-9) contains supplementary material, which is available to authorized users.

## Background

Microscopy techniques have been playing an important role in modern zoology. In recent decades, varieties of sectioning technologies have emerged to improve the quality of microscopic images [1–9], which can be divided into non-optical and optical strategies. Non-optical strategies include micro-computed tomography (Micro-CT)/nano-computed tomography (Nano-CT), magnetic resonance imaging (MRI), focus ion beam scanning electron microscopy (FIB-SEM)/serial block-face scanning electron microscopy (SBF-SEM) and so forth. Optical strategies involve confocal laser scanning microscopy (CLSM)/two-photon excitation microscopy (TPEM), selective plane illumination microscopy (SPIM), structured illumination microscopy (SIM) and so forth.

Micro-CT is an extensively used 3D imaging technique in zoology. It derived from computed tomography (CT), which has been used as a medical diagnostic tool since the early 1970s [10, 11]. With maximum spatial resolution of about 0.5 μm, it is routine to image hard and dry samples of approximately 1 ~ 200 mm in size. The imaging time usually takes a few hours. Other CT variants, such as Nano-CT, although capable of finer resolution (approximately 0.1 μm) and faster scanning speed (generally less than 1 h, e.g., 10 ~ 30 min), the size of sample should be in hundreds of micrometers. Based on the principle of nuclear magnetic resonance (NMR), MRI is predominantly used to measure the distribution of hydrogen protons within a sample [12], which makes MRI a technique ideally suited for imaging soft tissues, in many cases without using contrast agents. That is the reason why it is widely applicable to human body or organism containing plenty of water. FIB-SEM and SBF-SEM are block face imaging techniques in the family of SEM. With astonishing high resolution of approximately 4 ~ 7 nm, they are usually only suitable for samples in size of less than 1 mm. However, it would take many hours to obtain image stacks, and high-energy electrons may invade and damage the samples during the imaging process. For SBF-SEM there is a option to collect the sections of samples, while in FIB-SEM the sample would be pulverized.

In the field of light microscopy, CLSM and TPEM are the most regularly used techniques for obtaining high quality of optically sectioned images, which can be reconstructed and rendered in 3D by computers [5, 6, 13, 14]. However, both of them involve raster point scanning using an excitation laser source, leading to the drawbacks of complex configuration and being time-consuming. With a maximum resolution of approximately 0.2 μm, wide-field fluorescence microscopies with sectioning capability, including SPIM [7] and SIM [15–18], have recently met lots of interest due to the advantages of high spatial resolution, short image recording time and less photobleaching. SPIM utilizes a sheet beam to excite the fluorophores only in the small region around the in-focus plane. Since one sectioned slice can be obtained in only single shot, recording rapid process of animal’s retina and zebra-fish embryo have been reported and demonstrated by a few biological researchers [7, 19, 20]. However, because of the geometry of perpendicular detection against the sheet plane, they are not competent for non-transparent specimens. SIM is a wide-field technique in which a grid pattern is superimposed on the specimen while capturing images. The grid pattern is shifted in steps between the capture of each image set. The net result is the removal of out-of-focus fluorescence producing an increase in resolution beyond that of a general microscope, while allowing depth discrimination permitting 3D imaging. SIM has found numerous applications for time-lapse imaging of living tissues and cellular structures [21–23]. It was invented by Neil et al. [15] as a method of eliminating the out-of-focus background encountered in the wide-field microscopy. Furthermore, Gustafsson et al. [16–18] exploited the SIM to improve the spatial resolution based on a disparate theory, i.e. super-resolution SIM. In this paper, we only focus on the optical sectioning SIM.

**Fig. 1:**
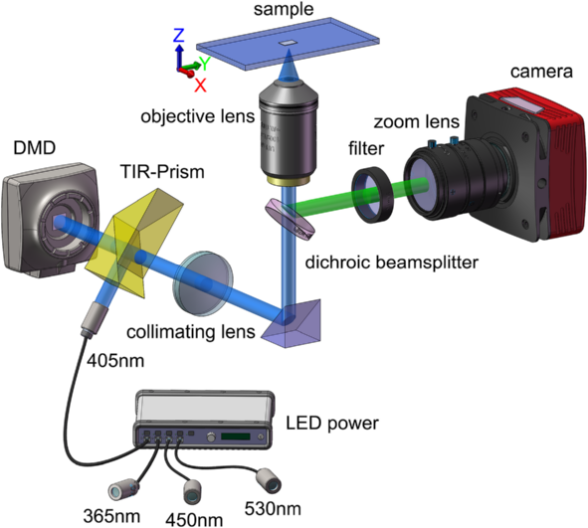
Schematic diagram of the DMD-based LED-illumination SIM microscope. For more specific information regarding components see ‘Methods’

We assess LED-SIM (DMD-based LED-illumination structured illumination microscopy, Fig. 1) for zoological studies of 3D structures in invertebrates, including roundworms, crustaceans, mites and insects. LED-SIM was first introduced by Dan et al. [24]. It has fast recording speed, high imaging quality and a compact and low cost configuration compared to the traditional SIMs. In LED-SIM, grid-pattern shifting is done exceptionally fast (i.e., 100Hz) so that imaging is essentially instant. This enables one to acquire and display optical sectioning images in real time [24]. The critical benefits of LED-SIM are that low illumination intensity light (1 w/cm^2^, less light than techniques such as CLSM) can be used, so fluorophores experience less photobleaching and standard fluorophores such as GFP or even scattering samples can be used. Thus LED-SIM is ideal for imaging of 3D structures in zoological studies. Here, we introduce LED-SIM and compare it to a few (not all of them) popular alternative techniques, involving conventional optical microscopy, CLSM and Micro-CT. We found that LED-SIM offered a distinct improvement over optical microscopes in terms of fast providing high-quality 3D images, as well as advantages over other advanced methods due to its affordability and ease of use. In addition, different preparation methods were also utilized to obtain better images according to the textures of the samples. For example, samples that have a tough cuticle (e.g., leg of flea beetle *Clavicornaltica* sp.) were cleared in H_2_O_2_ for better penetration, and samples with low autofluorescence (e.g., water flea *Daphnia* sp.) were stained with Congo red for imaging. Several slide mounting media were used, such as Hoyer’s medium (spermatheca of feathering beetle *Acrotrichis* sp.), glycerin (e.g., parasitic wasp *Aphitis* sp.) and methylcellulose (roundworm *Panagrellus redivivus*).

## Results

**Fig. 2:**
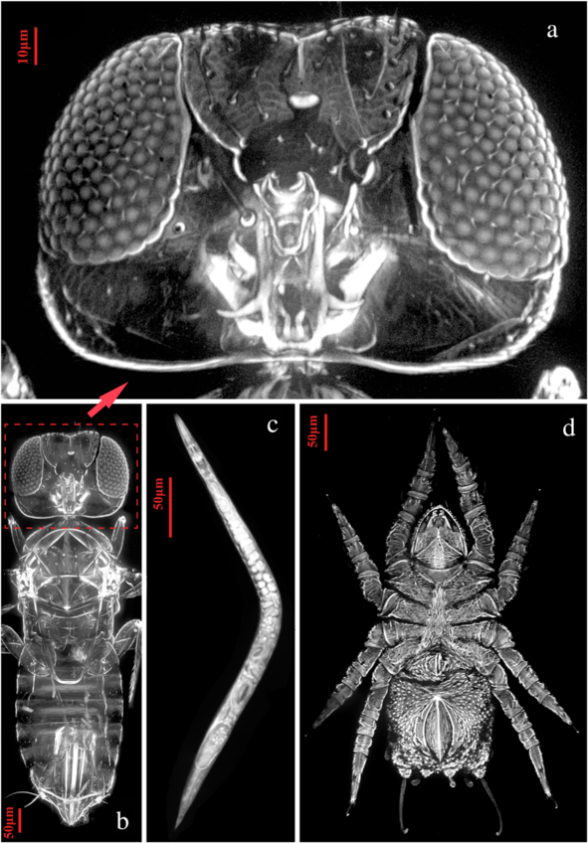
LED-SIM images of different small organisms. **a** Detail of the head of *Aphytis* sp. digitally zoomed from inset (**b**); **b** parasitic wasp *Aphytis* sp., a maximum-intensity projection image with 98 frames along the Z-axis under a 20× objective lens; **c** Roundworm *Panagrellus redivivus*, a maximum-intensity projection image with 26 frames along the Z-axis under a 20× objective lens; **d** Ventral view of a mite, a maximum-intensity projection image with 89 frames along the Z-axis under a 20× objective lens

**Fig. 3:**
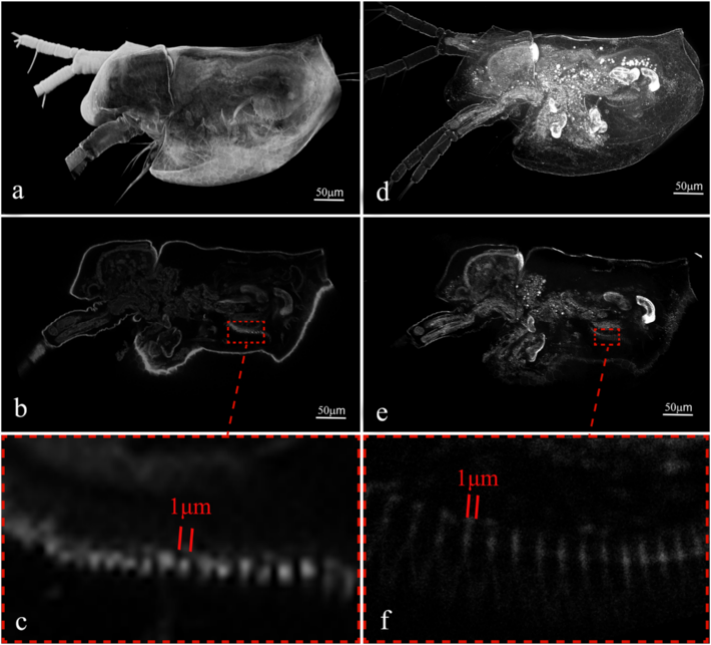
Comparison of the 3D images of a freshly hatched water flea *Daphnia* sp. obtained by CLSM (Leica TCS SP8) and LED-SIM. **a** CLSM image, a maximum-intensity projection of 150 frames along the Z-axis over a 147.2 μm thickness under a 25× objective lens (Additional file 1: Movie S1); **b** A single section obtained by CLSM; **c** A rectangular area digitally zoomed from inset (**b**). **d** LED-SIM image of the same sample, a maximum-intensity projection of 113 frames along the Z-axis under a 20× objective lens (Additional file 2: Movie S2); **e** A single section of obtained LED-SIM image; **f** A rectangular area digitally zoomed from inset (**e**)

**Fig. 4:**
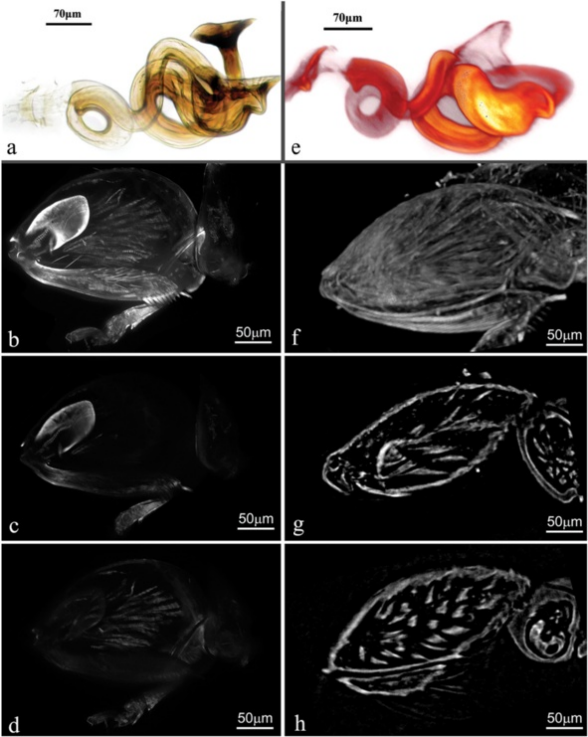
Comparison of LED-SIM image with Micro-CT and conventional optical microscope images. **a** Conventional microscopy image of spermatheca of feather-winged beetle *Acrotrichis* sp., stacks of images taken by a Zeiss Axiostar plus microscope and composed by Helicon Focus 6 software (Helicon Soft Ltd., Kharkov, Ukraine). **b** Maximum-intensity projection of a meta-femur of *Clavicornaltica* sp., obtained by LED-SIM with 3 subviews, 67 frames along the Z-axis under a 20× objective lens, obtained in 4 min, showing the spring tendon and muscle bundles inside the meta-femur. **c**, **d** Single sections of an obtained LED-SIM image. **e** LED-SIM image of spermatheca of *Acrotrichis* sp. (Additional file 3: Movie S3), generated by Amira 5.4.1 software. f Meta-femur of flea beetle *Clavicornaltica* sp., a maximum-intensity projection image of Micro-CT (MicroXCT-400, Carl Zeiss X-ray Microscopy, Inc., Pleasanton, USA) with 1000 frames along the Z-axis under a 40× objective lens, consumed approximately 24 h on scanning. **g**, **h** Single sections of obtained Micro-CT image

**Fig. 5:**
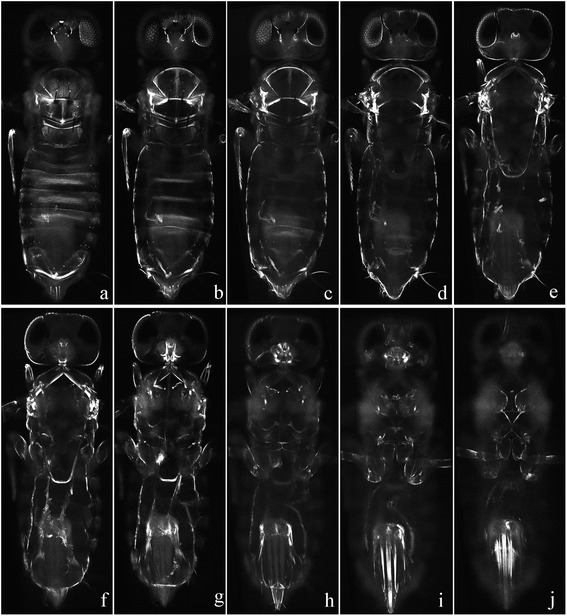
Consecutive section images of a parasitic wasp *Aphitis* sp. obtained by LED-SIM. Ten frames were selected from 98 frames along the Z-axis to present here, obtained under a 20× objective lens. The last three frames present strong interferences due to the thickness of the sample

All the samples we tested have proven to be suitable for LED-SIM imaging. Structures of these small organisms are clearly illustrated (Figs. 2, 3 and 4). All samples for LED-SIM imaging in current paper were under the excitation of 405 nm LED light and 20×/NA0.45 objective lens, which yielded a XY plane resolution of 500 nm and field-of-view (FOV) of 320 μm × 320 μm. The number of Z-stack images depended on the thickness of samples. The Z-scanning step was set to 200 nm. For large size samples, several subviews were required in order to stitch them into an extended FOV. Images obtained by CLSM, Micro-CT and conventional optical microscope are also provided as comparisons with LED-SIM imaging (Figs. 3 and 4). The time cost of imaging is the main advantage of LED-SIM, and its imaging speed is faster than other routine methods. The performances of LED-SIM and other techniques for each sample are as following:

Sample No. 1 (Fig. 2a, b; Fig. 5): *Aphytis* sp. (Insecta: Hymenoptera). Imaging system: LED-SIM. X-Y plane resolution: 500 nm. Z-stack of images: 98 frames, 5 subview stacks. Time of imaging: 3.35 min. Ten frames are selected from 98 frames along the Z-axis and presented in Fig. 5. The last three frames present strong interferences due to the thickness of the sample. Despite the weak cuticle of this sample, penetration depth achieved is only approximately 100 μm.

Sample No. 2 (Fig. 2c): *Panagrellus redivivus* (Nematoda: Panagrolaimidae). Imaging system: LED-SIM. X-Y plane resolution: 500 nm. Z-stack of images: 26 frames, 1 view-stack. Time of imaging: 15.85 s. Many details (e.g. pharynx, intestine, ovary, etc.) of *Panagrellus redivivus* with protein-abundant cuticle were shown clearly under LED-SIM (Fig. 2c), which indicates that this type of specimens are suitable for LED-SIM imaging.

Sample No. 3 (Fig. 2d): an unknown species of mite (Arachnida). Imaging system: LED-SIM. X-Y plane resolution: 500 nm. Z-stack of images: 89 frames, 6 subview-stacks. Time of imaging: 3.66 min. This mite species has strong cuticle, which limits light penetration, thus Fig. 2d presents very fine details on the surface of the specimen instead of internal structures.

Sample No. 4–1 (Fig. 3a-c; Additional file 1: Movie S1): *Daphnia* sp. (Crustacea, Cladocera). Imaging system: CLSM (Leica TCS SP8, Leica Inc., Solms, Germany). Light source: 561 nm. Objective lens: HCX IRAPO L 25×/0.95 WATER, Leica Inc., Solms, Germany. X-Y plane resolution: 1.213 μm. Z-stack of images: 150 frames. Time of imaging: approximately 15 min.

Sample No. 4–2 (Fig. 3d-f; Additional file 2: Movie S2): the same sample as Sample 4–1. Imaging system: LED-SIM. Z-stack of images: 113 frames, 12 subview-stacks. Time of imaging: 18.32 min. Compared to CLSM image, LED-SIM images present a better resolution along the lateral axis (with the highest resolution of 500 nm), but a slight loss of resolution along the Z-axis (with the highest resolution of 930 nm). It can be seen from the movie (Additional file 2: Movie S2) that both CLSM and LED-SIM fail to illustrate details when imaging depth exceeds approximately 100 μm.

Sample No. 5–1 (Fig. 4b-d): meta-femur of *Clavicornaltica* sp. (Insecta: Coleoptera). Imaging system: LED-SIM. X-Y plane resolution: 500 nm. Z-stack of images: 67 frames, 3 subview-stacks. Time of imaging: 4 min. Although the meta-femur of *Clavicornaltica* sp. is covered with extremely strong cuticle, the internal structures (e.g., muscles and metafemoral spring) can still be imaged with proper treatment (bleached in 30 % H_2_O_2_ in present sample).

Sample No. 5–2 (Fig. 4f-h): Meta-femur of *Clavicornaltica* sp. Imaging system: Micro-CT (MicroXCT-400, Carl Zeiss X-ray Microscopy, Inc., Pleasanton, USA). Light source: X-ray. Objective lens: 40×. X-Y plane resolution: 10 μm. Z-stack of images: 1000 frames. Time of imaging: approximately 24 h. A comparison of LED-SIM and Micro-CT images is presented in Fig. 4b-d, f-h.

Sample No. 6–1 (Fig. 4e, Additional file 3: Movie S3): spermatheca of *Acrotrichis* sp. (Insecta: Coleoptera). Imaging system: LED-SIM. X-Y plane resolution: 500 nm. Z-stack of images: 50 frames, 1 view-stack. Time of imaging: 40.49 s.

Sample No. 6–2 (Fig. 4a): the same sample as Sample 6–1. Imaging system: conventional microscope (Zeiss Axiostar plus, Zeiss Inc., Göttingen, Germany). Light source: visible light. Objective lens: (Zeiss A-Plan 20×/0.45, Zeiss Inc., Göttingen, Germany). X-Y plane resolution: approximately 1 μm. Z-stack of images: 20 frames. Time of imaging: approximately 10 min. A comparison of LED-SIM and conventional microscopy images is presented in Fig. 4a, c.

For LED-SIM imaging, various methods were utilized for the preparation of samples. All Arthropoda samples were absolute ethanol-preserved and the nematode was fixed in 4 % paraformaldehyde. Samples (No. 2, 3, 4) without high autofluorescence were stained with 0.15 % Congo red solution. Sample No. 1, 5 and 6 were not stained as they already exhibit strong autofluorescence under LED light (405 nm). Sample No. 5 with strong exoskeleton was bleached by 30 % H_2_O_2_ before mounting. Samples were mounted in glycerin, Hoyer’s medium or 3 % methylcellulose on a slide glass. The aim of using Hoyer’s mounting medium was to test whether this type of specimens are suitable for LED-SIM imaging, as a large number of museum-preserved specimens in modern zoological studies are mounted in Hoyer’s medium or neutral balsam (both with strong fluorescence), including valuable type specimens.

## Discussion

In the present study, there were only four different wavelengths of high-power LED light available (365 nm, 405 nm, 450 nm, and 530 nm). These four different wavelengths were all tested, and it turned out that 405 nm LED light is optimal for resilin-rich arthropod specimens. Thus it was employed for autofluorescent specimens in current study. Additionally, we found 405 nm LED light is also effectual for Congo red stained specimens when the optimal wavelength (561 nm [25]) is not available. The obtained images (Figs. 2, 3, 4 and 5) prove that the 405 nm LED light is a good excitation source for both Congo red stained and autofluorescent specimens. As 405 nm laser source is unavailable in our CLSM system, we used 561 nm laser light in CLSM imaging to yield best images for comparison with LED-SIM.

The comparison (Fig. 4a, e) of a LED-SIM image and a traditional optical image of the same spermatheca of a feather-winged beetle indicated that the LED-SIM technique had the ability to visualize internal structures of small organisms with good quality. LED-SIM, with higher resolution, showed more clear and distinct 3D structures, which can be important to taxonomists studying reproductive structures in small specimens. Many “non-destructive dissections” and “virtual dissections” [26] of slide-mounted specimens could be accomplished by LED-SIM. The spermatheca sample (with good autofluorescence) we use in this paper was mounted in Hoyer’s medium without staining. Most of the museum slides containing preserved structures or specimens were prepared in a similar way. LED-SIM could be useful to generate 3D computer images of valuable specimens that mounted in Hoyer’s medium, glycerin, or neutral balsam.

Most LED-SIM images we obtained presented a good resolution along the lateral axis (with the highest resolution of 500 nm), but with a slight loss of resolution along the Z-axis (with the highest resolution of 930 nm). Similar results were found in a previous study comparing commercial CLSM and SIM devices [27]. The suitable size (currently limited by the objective lens) for samples is less than one centimeter. Preferable thickness of samples is less than 100 μm. Although Dan et al. (2013) [24] found that a 200 μm penetration depth was reached at the imaging of the Golgi-stained neuron cells, our results demonstrated that LED-SIM images presented strong interference when the sample thickness exceeded approximately 100 μm (e.g., Fig. 5). This may be due to the property of the samples that we used, most of which have strong cuticles and hard internal structures. Not only the property of samples, but also the mounting media and wavelength of LED light are important factors that could influence the penetration depth. Generally, LED-SIM is not able to compete with many techniques (such as SPIM, LSFM and OPT) in respect of penetration depth, but LED-SIM has preferable resolution in terms of lateral axis. Meanwhile, LED-SIM still has insufficient axial resolution compared to some other techniques, such as SIM microscopy using three beams for interference illumination [20, 23, 28] or SR-SIM combining ultrathin planar illumination produced by scanned Bessel beams [29]. Dan et al. (2013) [24] proposed to combine a micro-optical sectioning tomography (MOST) system [30] with a LED-SIM system, in which case a nearly isotropic sub-micron spatial resolution in all three dimensions can be achieved.

As visualization becomes increasingly important in functional and evolutionary studies [31], resolution is one of the most important features of imaging equipment, as well as related technical features such as imaging speed, data size, and penetration depth. The resolution of 500 nm in the XY-plane and 930 nm along the Z-axis was achieved with a 20× objective lens (Plan Fluor ELWD 20×/NA0.45, Nikon Inc., Tokyo, Japan) in the current study, while the finest XY-plane resolution of LED-SIM could approach 90 nm with a 100× objective lens (Apo TIRF, NA1.49, Nikon Inc., Tokyo, Japan) [24]. The imaging speed of a complete stack of images (for 3D representation) ranged from tens of seconds to a few minutes and was dependent on the resolution and the number of frames required.

## Conclusion

The conventional optical microscopy is remains the imaging system that most researchers utilize, due to its affordability for small laboratories or small research groups. However, conventional microscopes have difficulties in visualizing internal structures of biological samples. Three-dimensional imaging is now possible due to innovative imaging techniques, as well as powerful computers and software to generate 3D renderings. The insights gained from these images have advanced knowledge throughout the life sciences.

Research in many directions in comparative zoology has seen a surge in activity recently, enabled by the innovation of microscopic methods and techniques. These applications of innovative techniques include Micro-CT, CLSM, MRI, etc. (Table 1). LED-SIM can neither provide extraordinarily fine lateral resolution (such as FIB-SEM does) nor perfect isotropic resolution (such as Micro-CT does), and there are also difficulties in imaging of samples that exceed 100 μm in thickness. However, it might offer advantages for imaging of fluorescent signals (either labelled signals or autofluorescence) in small animal samples due to its affordability and ease of use.

**Table 1:** Comparison of the features of different major 3D imaging systems used in zoological studies. The features of the different methods are based on our knowledge of the different pieces of equipment established by our previous studies; various statistics from recent papers are also referenced

	Lateral resolution	Suitable sample type	Estimated cost (US$)	Suitable sample size	Imaging light source	Sample preparation	Imaging time	Imaging color
LED-SIM	0.1 μm	Dry or wet	~200,000	<10 mm	LED	Fast	<10 min	Pseudo color
Laser-SIM	0.1 μm	Dry or wet	550,000–650,000	<10 mm	Laser	Fast	<10 min	Pseudo color
CLSM	0.2 μm	Dry or wet	500,000–700,000	<10 mm	Laser	Medium	30 min–1 h	Pseudo color
SPIM	0.2 μm	Dry or wet	300,000–500,000	<10 mm	Laser	Medium	<10 min	Pseudo color
Micro-CT	0.5–5 μm	Dry or wet	500,000–700,000	1–200 mm	X-ray	Medium	2–24 h	Black and white
FIB/SBF-SEM	4–7 nm	Dry	800,000–900,000	<1 mm	Electron and ion beam	Medium	8–9 h	Black and white
MRI	20 μm	Wet	600,000–800,000	10–50 mm	Radio frequency	Medium	~24 h	Pseudo color

The remaining challenges for LED-SIM include providing more options of LED wavelength, advanced optimization and simplification of the imaging apparatus. Although the axial resolution of LED-SIM is currently not considered to be high, it hopefully will be improved by combining LED-SIM with other techniques (e.g., optical sectioning tomography technology).

## Methods

### Sample preparation

All specimens used in this study are housed in the Institute of Zoology of the Chinese Academy of Sciences (IZAS).

Sample No. 1 (Fig. 2a, b; Fig. 5). Scientific name: *Aphytis* sp. (Insecta: Hymenoptera). Specimens were collected from field in Fuzhou, Fujian, China. Sample preparation: absolute ethanol-preserved specimens were selected, without staining or H_2_O_2_ bleaching, and then mounted in glycerin on a slide glass; subsequently, the margins of the cover glass were sealed by nail polish.

Sample No. 2 (Fig. 2c). Scientific name: *Panagrellus redivivus* (Nematoda: Panagrolaimidae). Specimens were obtained from an aquarium company in Shijiazhuang, Hebei, China. Sample preparation: live specimens were selected and fixed in 4 % paraformaldehyde for 1 h, stained in 0.15 % Congo red solution for approximately 5 min, and then mounted in 3 % methylcellulose (prevents soft samples from shrinking) on a slide glass; subsequently, the margins of the cover glass were sealed by nail polish.

Sample No. 3 (Fig. 2d). Scientific name: unknown species of mite (Arachnida). Specimens were collected from the Qinling Mountains, Shaanxi, China. *Sample preparatio*n: absolute ethanol-preserved specimens were selected, stained in 0.15 % Congo red solution for approximately 5 min, and then mounted in glycerin on a slide glass; subsequently, the margins of the cover glass were sealed by nail polish.

Sample No. 4–1 (Fig. 3a-c), Sample No. 4–2 (Fig. 3d-f). Scientific name: *Daphnia* sp. (Crustacea, Cladocera). Specimens were obtained from an aquarium company in Putian, Fujian, China. Sample preparation: freshly hatched specimens were selected and then killed in absolute ethanol, stained in 0.15 % Congo red solution for approximately 5 min, and mounted in glycerin on a slide glass; subsequently, the margins of the cover glass were sealed by nail polish.

Sample No. 5–1 (Fig. 4b-d). Meta-femur of *Clavicornaltica* sp. (Insecta: Coleoptera). Specimens were collected from a field in Fuan, Fujian, China. Sample preparation: absolute ethanol-preserved specimens were selected, the meta-femurs were carefully torn off, without staining, steeped in 30 % H_2_O_2_, heated for a few minutes to bleach the cuticle, washed in distilled water for several times, and then mounted in glycerin on a slide glass; subsequently, the margins of the cover glass were sealed by nail polish.

Sample No. 5–2 (Fig. 4f-h). Meta-femur of *Clavicornaltica* sp. (Insecta: Coleoptera). Specimens were collected from a field in Fuan, Fujian, China. Sample preparation: absolute ethanol-preserved specimens were selected, the meta-femurs were carefully torn off and dried at the critical point (hcp-2, Hitachi Inc., Tokyo, Japan), and then glued to the tip of a micropipette using nail polish.

Sample No. 6–1 (Fig. 4e), Sample No. 6–2 (Fig. 4a). Spermatheca of *Acrotrichis* sp. (Insecta: Coleoptera). Specimens were collected from the Qinling Mountains, Shaanxi, China. Sample preparation: absolute ethanol-preserved specimens were selected, and the spermatheca were directly mounted in Hoyer’s medium on slide glass after dissection; subsequently, the margins of the cover glass were sealed by nail polish.

Congo red staining: The formulation of Congo red solution followed Michels and Buentzow (2010; 1.5 mg per milliliter of distilled water) [25]. After staining, the samples were washed in distilled water until no residual Congo red was present. The preparation and formulation of Hoyer’s medium followed Van der Meer (1977) [32].

These six different materials were selected from different invertebrate clades to represent a broad set of samples: e.g., roundworms (Nematoda) with protein-abundant cuticle and arthropods (Arthropoda) with cuticle dominated by chitin. First, amongst the arthropods, flea beetles (Insecta, Coleoptera, *Clavicornaltica* sp.) and mites (Arachnida, Acarina, unknown species) were selected to represent specimens with strong cuticle and limited light penetration. Second, water fleas (Crustacea, Cladocera, *Daphnia* sp.) and parasitoid wasps (Insecta, Hymenoptera, *Aphytis* sp.) were selected to represent specimens with weak cuticle and fragile internal structures. Third, a spermatheca of a feather-winged beetle (Insecta, Coleoptera, *Acrotrichis* sp.) was selected to represent fine internal structures of invertebrates.

### Imaging systems and image processing

The LED-SIM system (Xi’an Institute of Optics and Precision Mechanics, Chinese Academy of Sciences) used in the current paper is illustrated in Fig. 1. A wavelength-switchable and high-power LED (UP314, UVATA Inc., China) was used as the light source. A digital micro-mirror device (DMD) (1024 × 768 pixels, V-7000UV, ViALUX GmbH, Germany) modulated the incident light into structured sinusoidal patterns, which were then projected on the sample via an objective lens (Plan Fluor ELWD 20×/NA0.45, Nikon Inc., Tokyo, Japan). Fast phase-shifting was realized by refreshing the DMD chip. Through a 405 nm longpass filter, the excited fluorescence light was recorded by a highly dynamic and sensitive sCMOS camera (ORCA Flash 4.0, 2048 × 2048 pixels @ 100 fps, Hamamatsu Inc., Tokyo, Japan). In front of the camera, a zoom lens (70–300 mm, F/4-5.6, Nikon Inc., Tokyo, Japan) and an extender lens (N-AF 2× Teleplus MC4, Kenko Inc., Tokyo, Japan) were incorporated with the objective lens to produce variable magnification of the image. The sample was mounted on a manual XY and motorized Z-axis translation stage (M-405.PG, Physik Instrumente Inc., Karlsruhe, Germany) that was able to scan axially in minimum step of 50 nm. Automated data collection, DMD pattern generation, and motorized stage movement were implemented by custom software programmed in C++.

A commercial laser confocal system Leica TCS SP8 (Leica Inc., Solms, Germany) and a Micro-CT system MicroXCT-400 (Carl Zeiss X-ray Microscopy, Inc., Pleasanton, USA) (both in the Institute of Zoology, Chinese Academy of Sciences) were used to capture 3D images for comparison to LED-SIM.

Amira 5.4.1 (Visage Imaging, San Diego, USA) and Imaris 7.2.1 (Bitplane Inc., Zurich, Switzerland) software were used to produce 3D images. In addition, a conventional optical imaging system consisting of a Zeiss Axiostar plus microscope (Zeiss Inc., Göttingen, Germany), Nikon D300 digital camera (Nikon Inc., Tokyo, Japan) and Helicon Focus 6 software was used to capture and compose 2D images. The final figures were prepared with Photoshop CS5 (Adobe, San Jose, USA) and Illustrator CS5 (Adobe, San Jose, USA).

## Additional files

Additional file 1: Movie S1. 3D animation of the structures of a freshly hatched water flea *Daphnia* sp. obtained by CLSM (for comparison with LED-SIM). (MP4 8973 kb) Additional file 2: Movie S2. 3D animation of the structures of a freshly hatched water flea *Daphnia* sp. (the same sample used in movie S1) obtained by LED-SIM. (MP4 9282 kb) Additional file 3: Movie S3. 3D animation of the spermatheca of feather-winged beetle *Acrotrichis* sp. obtained by LED-SIM. (MP4 19818 kb)
